# Weighted gene correlation network analysis reveals novel biomarkers associated with mesenchymal stromal cell differentiation in early phase

**DOI:** 10.7717/peerj.8907

**Published:** 2020-04-03

**Authors:** Bin Xiao, Guozhu Wang, Weiwei Li

**Affiliations:** Department of Orthopedics, Second Affiliated Hospital of Shaanxi University of Traditional Chinese Medicine, Xianyang, Shaanxi, China

**Keywords:** WGCNA, Mesenchymal stem cells, Cell differentiation, Biomarkers, Hub genes, Bioinformatics

## Abstract

Osteoporosis is a major public health problem that is associated with high morbidity and mortality, and its prevalence is increasing as the world’s population ages. Therefore, understanding the molecular basis of the disease is becoming a high priority. In this regard, studies have shown that an imbalance in adipogenic and osteogenic differentiation of bone marrow mesenchymal stem cells (MSCs) is associated with osteoporosis. In this study, we conducted a Weighted Gene Co-Expression Network Analysis to identify gene modules associated with the differentiation of bone marrow MSCs. Gene Ontology and Kyoto Encyclopedia of Genes and Genome enrichment analysis showed that the most significant module, the brown module, was enriched with genes involved in cell cycle regulation, which is in line with the initial results published using these data. In addition, the Cytoscape platform was used to identify important hub genes and lncRNAs correlated with the gene modules. Furthermore, differential gene expression analysis identified 157 and 40 genes that were upregulated and downregulated, respectively, after 3 h of MSCs differentiation. Interestingly, regulatory network analysis, and comparison of the differentially expressed genes with those in the brown module identified potential novel biomarker genes, including two transcription factors (ZNF740, FOS) and two hub genes (FOXQ1, SGK1), which were further validated for differential expression in another data set of differentiation of MSCs. Finally, Gene Set Enrichment Analysis suggested that the two most important candidate hub genes are involved in regulatory pathways, such as the JAK-STAT and RAS signaling pathways. In summary, we have revealed new molecular mechanisms of MSCs differentiation and identified novel genes that could be used as potential therapeutic targets for the treatment of osteoporosis.

## Introduction

Osteoporosis is a systemic metabolic bone disease characterized by reduction in bone mass and degeneration of bone microstructure, which makes the bone brittle and prone to fracture. Osteoporosis has become an important public health problem as the global population ages. Therefore, revealing the molecular mechanisms of osteoporosis and developing effective and preventive treatments would be crucial to the human well-being. Bone remodeling occurs at discrete sites called bone remodeling units, wherein mineralized bone resorption occurs by osteoclasts that break down the tissue in bones and release the minerals to the blood. Osteoblasts are then recruited to the site after osteoclasts undergo apoptosis, and a new bone is eventually formed (Compston, McClung & Leslie, 2019). The imbalance between bone resorption and bone formation can lead to various diseases, such as osteopenia, osteopetrosis and osteoporosis. While osteoclasts are derived from hematopoietic stem cell precursors, osteoblasts and adipocytes are derived from bone marrow mesenchymal stem cells (MSCs) (Chen et al., 2016b). MSCs are mesoderm-derived multipotent cells that adhere to culture dish surface and can proliferate and differentiate into adipocytes, osteoblasts and chondrocytes in vitro. Aging is associated with reduced osteogenic (OS) potential and increased fat formation by MSCs, which can lead to senile osteoporosis (Bergman et al., 1996).

With the advent of the era of big data, a variety of biological public databases have emerged, which provide a large amount of accessible genomic and clinical data for basic and translational studies. Today, the Gene Expression Omnibus (GEO) database is considered the most comprehensive public repository of large-scale genomics data. These databases allow for mining meaningful genomic changes and for discovering biological mechanisms that are involved in disease development and progression (Gruebner et al., 2017). In addition, mining public databases can provide insights into the functional networks of unknown genes, and thus, confirming gene expression trends that may be overlooked in a single experiment. Besides, large-scale analysis can validate experimental findings, provide supportive evidence, help design a research plan, and test an already set hypothesis (Lachmann et al., 2018).

Weighted Gene Co-Expression Network Analysis (WGCNA) is a widely used technique for transcriptomic data analysis (Langfelder & Horvath, 2008). It is a new systems biology approach that is frequently used to study the time course of cell differentiation and biological development (Li et al., 2017; Liu et al., 2017; Tao et al., 2018). WGCNA can be used to construct gene expression networks by clustering highly correlated genes into modules. These biologically relevant modules usually contain functionally related genes and may include key driver genes that can serve as potential diagnostic and prognostic biomarkers, or as therapeutic targets (Chen et al., 2016a; Chou et al., 2014).

In this study, we constructed a WGCNA network using expression data of MSCs during OS and adipogenic (AD) differentiation. Biological enrichment analysis on modules of interest and their corresponding hub genes identified potential key driver genes that could be used as biomarkers and therapeutic targets of osteoporosis.

## Materials and Methods

### Expression analysis of microarray data

The GSE80614 data set (S4–S6) was downloaded from the GEO Database (Van de Peppel et al., 2017). In this data set, total RNA was obtained from bone marrow MSCs that were cultured in an OS medium or in an adipose differentiation medium. Data analysis was performed in an R environment (R Core Team, 2018). The *limma* package (http://www.bioconductor.org/packages/release/bioc/html/limma.html) was used for differentially expressed genes (DEGs) analysis (S7 and S9). A cut-off of log2 Fold Change (log2FC) > 1 and an adjusted *P*-value < 0.05 was used to extract biologically meaningful genes (Ritchie et al., 2015).

### Co-expression network construction

Briefly, the WGCNA R package was used for network construction (S8). Pair-wise Pearson correlation between each pair of genes was first estimated to identify highly correlated genes with consistent profiles across samples. The adjacency matrix was then converted to a topological overlap matrix (TOM) to identify gene modules and highly correlated gene clusters. Each TOM was subsequently used as an input file to perform hierarchical clustering analysis using the function *flashClust* (Ponsuksili et al., 2013).

### Module-trait relationships

The correlation between modules eigengenes and clinical traits was used to estimate module-trait relationship. Modules that were significantly correlated with biological and clinical variables were selected for subsequent analysis. Gene significance (GS) was also evaluated using the absolute correlation of gene expression profiles with biological and clinical variables. On the other hand, module membership (MM) of a gene was defined as the correlation between the gene expression profile and a module’s eigengene (Horvath & Dong, 2008).

### Enrichment analysis of the identified modules

Gene Ontology (GO, http://www.geneontology.org) is a knowledge base used for annotating genes, gene products and gene sequences as potential biological phenomena (2015). The Kyoto Encyclopedia of Genes and Genome (KEGG, https://www.kegg.jp/) is a comprehensive database for the biological interpretation of genomic and other high-throughput data (Kanehisa & Goto, 2000). The database for annotation, visualization and integrated discovery (DAVID) is an annotation tool accounting for over 80% of the overall functional enrichment portal usage; however, it has not been updated since 2016 (Dennis et al., 2003). On the other hand, compared with DAVID, Metascape provides a more frequently updated bioinformatics platform (Zhou et al., 2019). Thus, in order to investigate the underlying relation between gene modules and relevant clinical traits in this study, the online bioinformatics database Metascape (http://metascape.org) was used to analyze the overrepresentation of genes from selected modules with biological process GO terms, KEGG pathways, Reactome gene sets, Canonical Pathways and Comprehensive Resource Of Mammalian Protein Complexes (CORUM). Enriched terms with *P* ≤ 0.01 were considered significant.

### Identification of genes involved in MSC differentiation

A protein–protein interaction (PPI) network of 608 genes from the brown module was obtained using the Metascape website. The Cytoscape (version 3.6.1, https://cytoscape.org/) plug-in, Molecular Complex Detection (MCODE, http://apps.cytoscape.org/apps/mcode), was used to detect prominent genes in this PPI network (Bandettini et al., 2012; Shannon et al., 2003). The following analysis parameters were used as the cut-off criteria: Degree = 2, node score = 0.2, *k*-core = 2 and max depth = 100 (Bader & Hogue, 2003). In addition, biologically relevant genes in the brown module were detected by comparing the brown module with DEGs during MSC differentiation using a Venn diagram web-tool (http://bioinformatics.psb.ugent.be/webtools/Venn/).

### Transcription factors and miRNAs regulatory network

The regulatory mechanisms within modules were predicted using public databases. Upstream regulatory miRNAs were explored using miRNA databases, including TargetScan (http://www.targetscan.org/vert_72), miRTarBase (http://mirtarbase.cuhk.edu.cn/php/index.php) and miRDB (http://mirdb.org). The resulted networks were constructed using Cytoscape (Deng et al., 2017; Hsu et al., 2011; Riffo-Campos, Riquelme & Brebi-Mieville, 2016; Wong & Wang, 2015). Similarly, upstream transcription factors were detected using the iRegulon (http://apps.cytoscape.org/apps/iregulon) plugin in Cytoscape (Janky et al., 2014). In fact, iRegulon can analyze the enrichment of transcription factor motifs in target sequences using a position matrix method. Briefly, the string website (https://string-db.org) was first used to construct a PPI network of hub genes (Von Mering et al., 2005). The network was then imported into Cytoscape and analyzed with iRegulon using the relevant parameters. iRegulon determines the optimal subset of direct target genes of transcription factors according to the motifs or tracks methods. The minimum identity between orthologous genes was set to 0.05 and the maximum false discovery rate on motif similarity was set to 0.001. Correlations with Normalized Enrichment Score (NES) > 5.0 were selected for further analysis.

### Validation of candidate genes

The E-MEXP-858 data set from the ArrayExpress database (https://www.ebi.ac.uk/arrayexpress) was used to validate the expression profile of the identified hub genes. DEGs analysis was performed by comparing MSCs during differentiation between 0 and 1 h, and between 0 and 3 h.

### Gene set enrichment analysis

The GSEA (http://software.broadinstitute.org/gsea/index.jsp) is a computational method for interpreting genome-wide expression profiles using the following three key elements: the calculation of enrichment scores (ES), the estimation of the significance level of ES, and the adjustment for multiple hypothesis testing (Subramanian et al., 2005). GSEA was performed to elucidate key pathways involved in high vs low gene expression groups. A nominal *P*-value < 0.05, a false discovery rate (FDR) < 0.05, and | NES | ≥ 1 were used to identify significant pathways.

## Results

### Co-expression network construction

**Figure 1:**
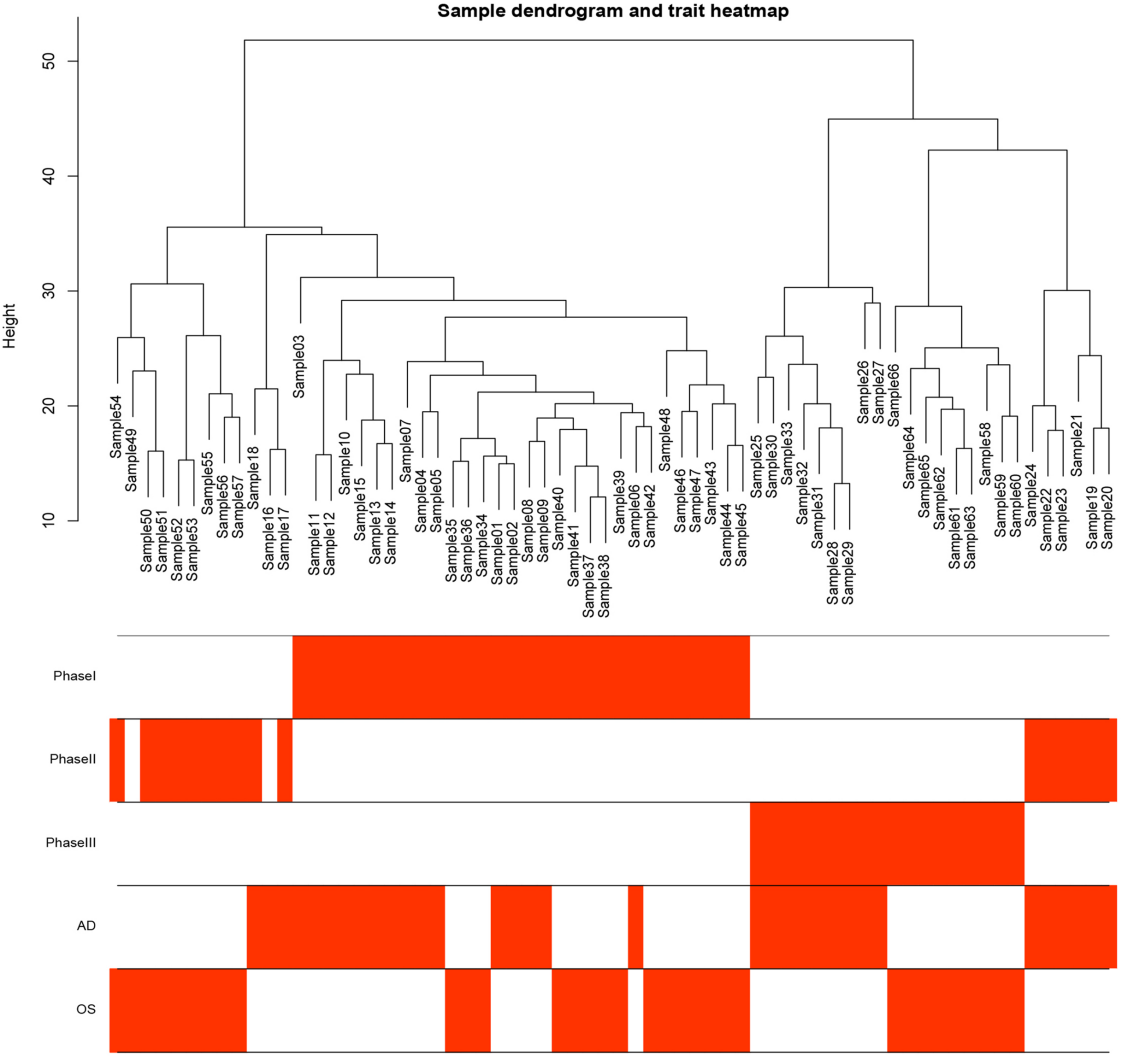
Samples tree and traits heat map. The leaves of the tree correspond to bone marrow MSCs AD samples, OS samples and undifferentiated samples. Bands 1–3 below the tree represent differentiation between Phase I, Phase II and Phase III. Bands 4–5 represent AD and OS differentiation of bone marrow MSCs, respectively. MSCs, mesenchymal stem cells; AD, adipogenic; OS, osteogenic; Phase I: 0–3 h, Phase II: 48–96 h, Phase III: 48–96 h.

**Figure 2:**
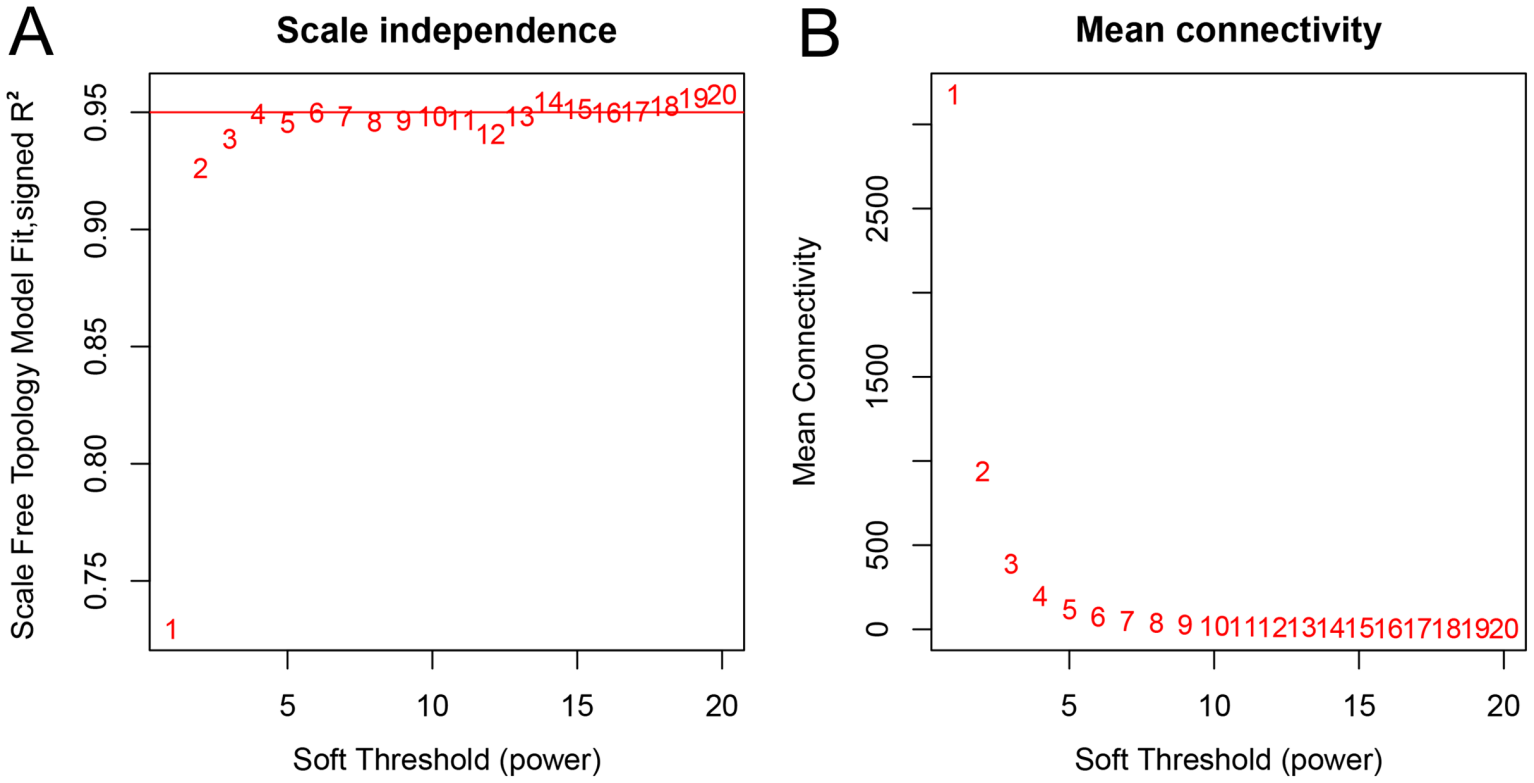
Network topology analysis of various soft threshold powers. (A) Scale-free fit index, with the signed *R*^2^ (*y*-axis) and the soft threshold power (*x*-axis). Select β = 7 was used for subsequent analysis. (B) The average connectivity (*y*-axis) is a strictly decreasing function of power β (*x*-axis).

**Figure 3:**
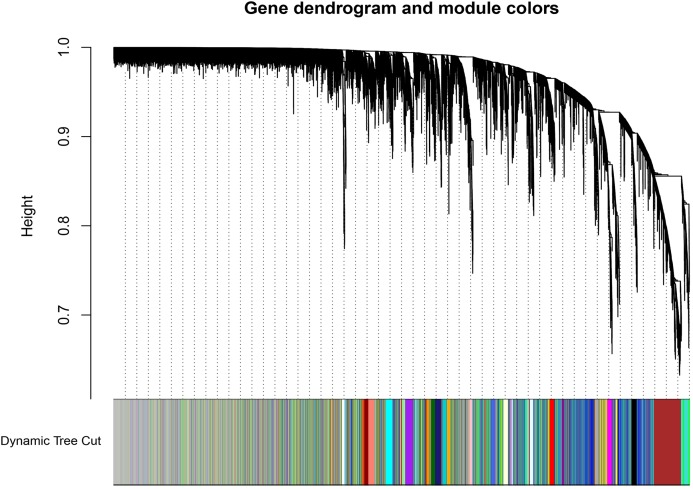
Dendrogram of gene expression. The color annotations provide a simple visual comparison of module assignments (branch cuttings) based on the dynamic tree cutting method.

A total of 66 samples and 19,610 genes from the GSE80614 data set were used for network construction. One of the most critical parameters in WGCNA network construction is the power value, which affects the independence and average connectivity of the co-expression modules. The degree of independence reached 0.95 and the average connectivity was highest using a power equal to 7 (Figs. 1 and 2). The weighted network was then constructed based on the scale-free topology criteria. Eventually, 22 modules were detected using the dynamic tree cutting approach (Fig. 3).

### Gene co-expression modules were associated with clinical traits

**Figure 4:**
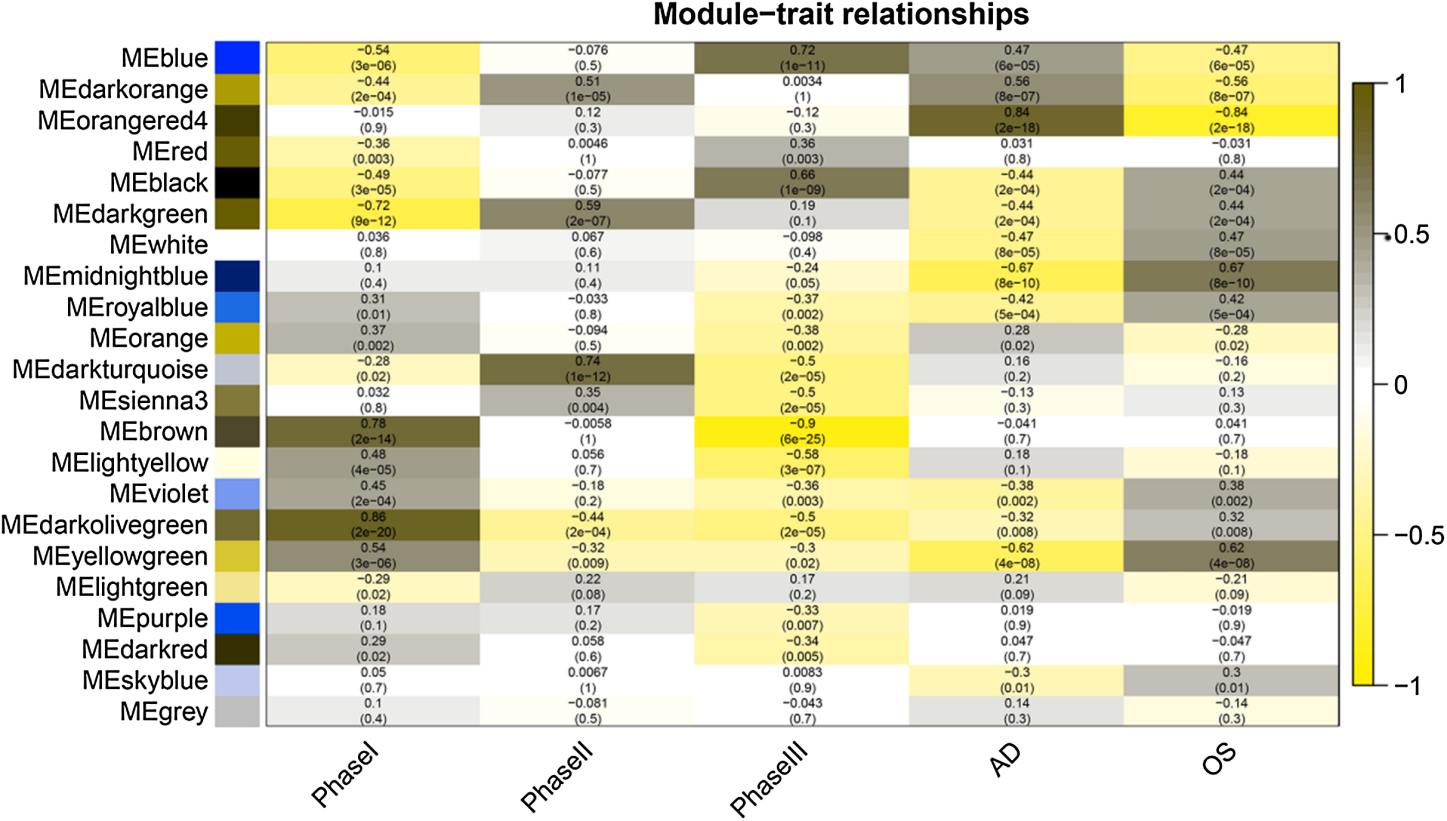
Module-trait relationships. Phase I: 0–3 h, Phase II: 6–24 h, Phase III: 48–96 h, AD, samples of adipogenic differentiation, OS, samples of osteogenic differentiation.

**Figure 5:**
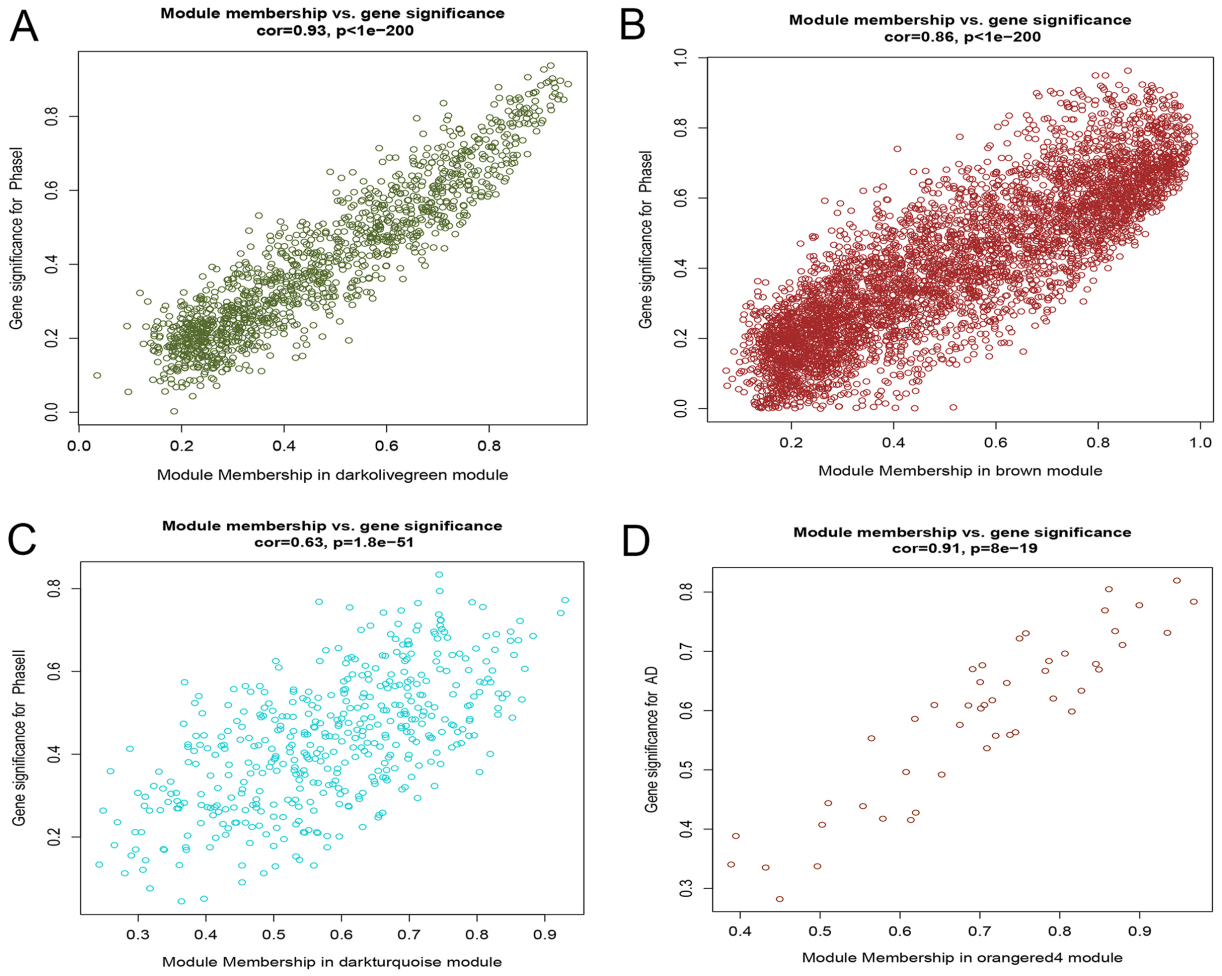
Scatter diagram of MM against GS. (A) Scatter diagram of MM of the darkolivegreen module genes against GS with Phase I differentiation. (B) Scatter diagram of MM of the brown module genes against their GS with Phase I differentiation. (C) Scatter diagram of MM of the darkturquoise module genes vs GS with Phase II differentiation. (D) Scatter diagram of MM of the orangered4 module genes vs GS with AD differentiation. MM: module membership, GS, gene significance; Phase I: 0–3 h, Phase II: 6–24 h, AD, adipogenic.

**Figure 6:**
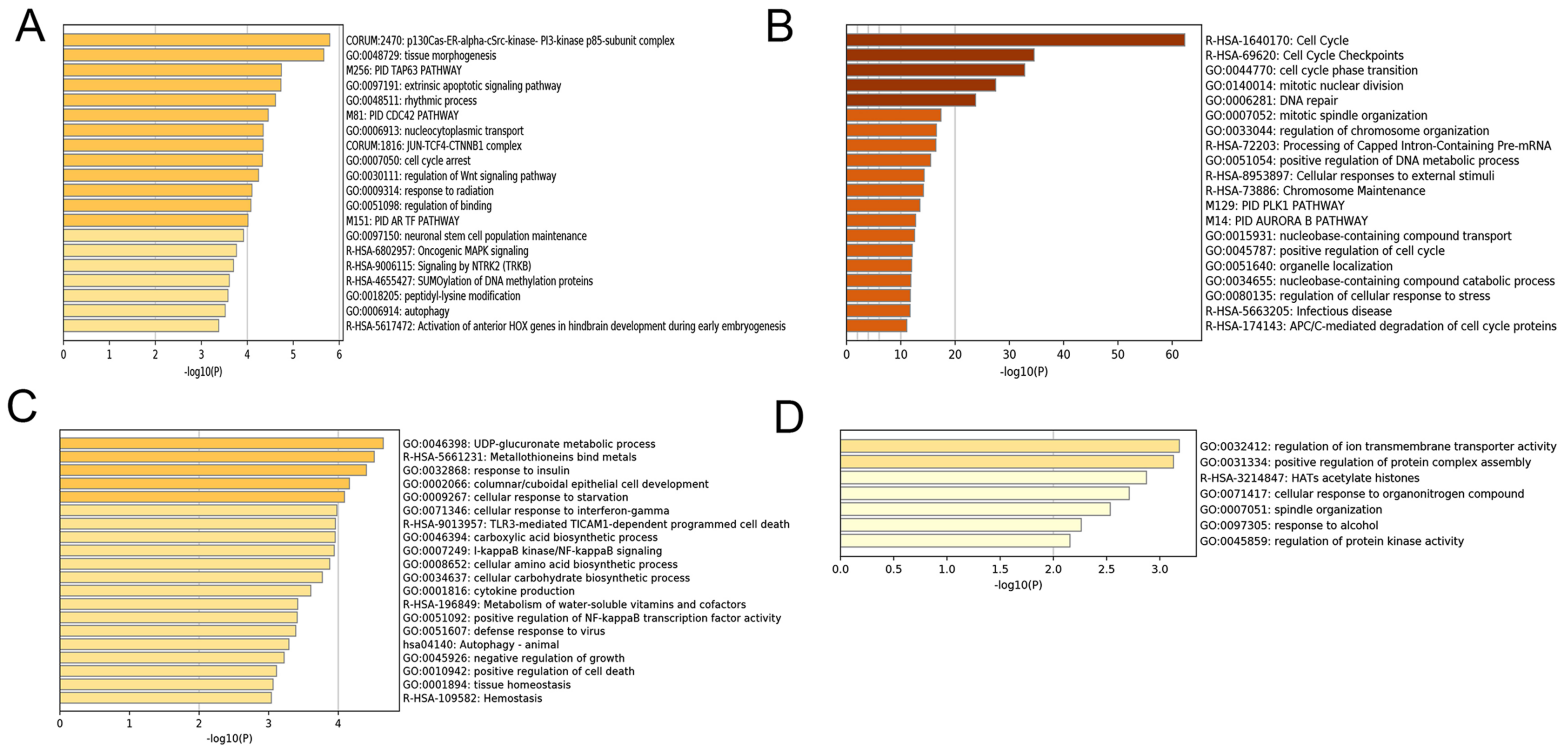
Enrichment analysis of selected modules. (A–D) Heat maps of the top 20 enriched terms for DEGs in the darkolivegreen, brown, darkturquoise and orangered4 modules, respectively. Colors represent *P*-values. DEGs, differentially expressed genes.

After correlating the modules with clinical traits, a high correlation was observed with Phase I, Phase II and AD differentiation stages of mesenchymal cells (Fig. 4). The darkolivegreen module (*r* = 0.86, *P* = 2e^−20^) had the highest correlation with Phase I, followed by the brown module (*r* = 0.78, *p* = 2e^−14^). The darkturquoise module (*r* = 0.81, *p* = 3e^−16^) had the highest correlation with Phase II of differentiation. On the other hand, the orangered4 module had the highest correlation (*r* = 0.84, *p* = 2e^−18^) with the AD phase. The inter- and intra-modular gene correlation of the four modules was then plotted based on GS and MM (Figs. 5A–5D). GO and KEGG enrichment analysis was then performed, which showed that 893 genes from the darkolivegreen module were mainly enriched for the p130Cas-ER-alpha-cSrc-kinase-PI3-kinase p85-subunit complex and tissue morphogenesis (Fig. 6A, *P* < 0.01). The 2,879 genes in the brown module were primarily enriched for Cell Cycle and Cell Cycle Checkpoints (Fig. 6B, *P* < 0.001). On the other hand, 437 genes in the darkturquoise module were associated with the UDP-glucuronate metabolic process and with the term “Metallothioneins bind metals” (Fig. 6C, *P* < 0.01). Finally, 47 genes of the orangered4 module were principally enriched for leukocyte apoptotic processes and the regulation of ion transmembrane transporter activity (Fig. 6D, *P* < 0.01).

### Identification of module-related hub genes and lncRNAs

**Figure 7:**
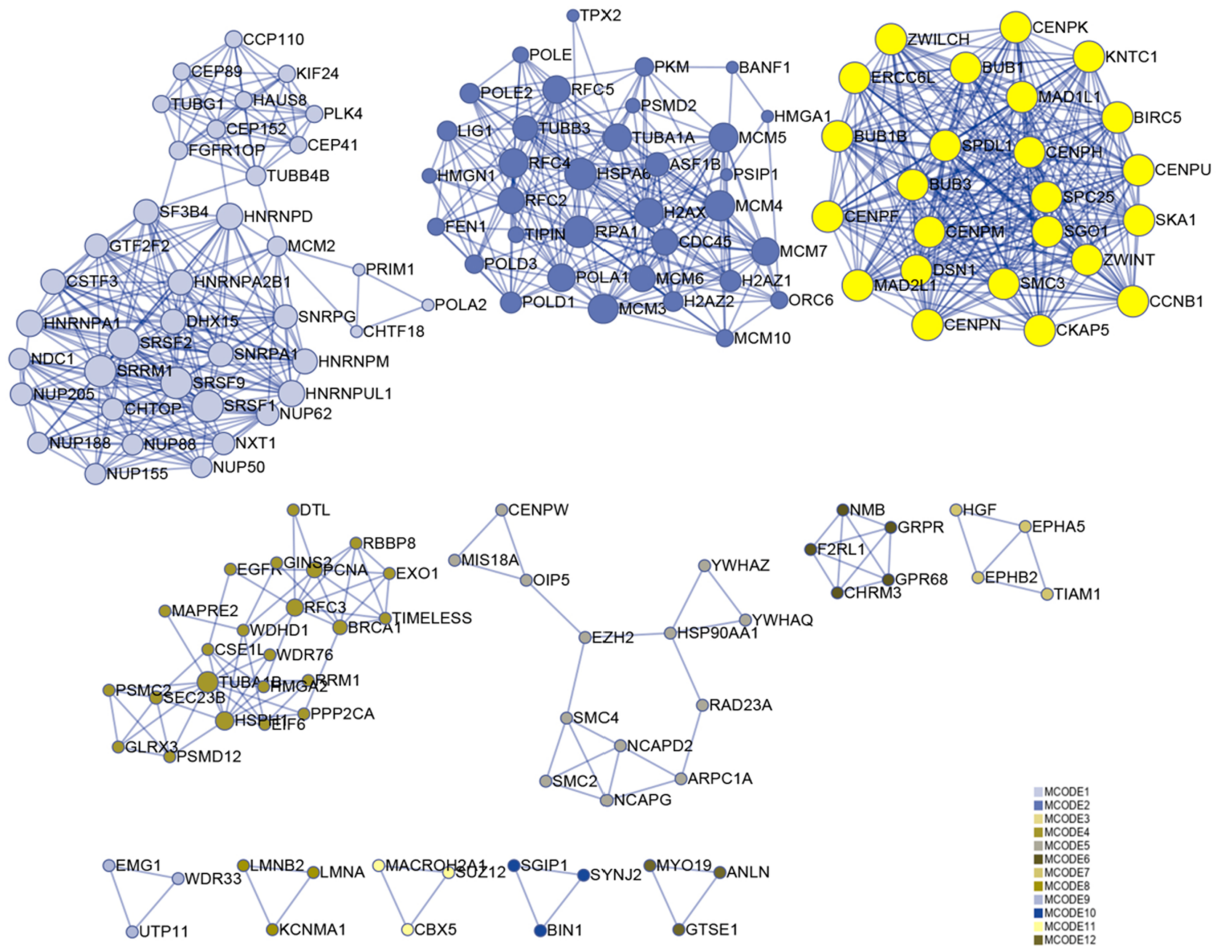
Functional enrichment analysis. A total of 608 genes from the brown module were analyzed for their enrichment with GO terms and KEGG pathways. After analysis on the Metascape website, the hub module was selected using the MCODE plugin of Cytoscape software (MCODE score = 23, nodes = 24 and edges = 276), in which the yellow color molecules represent the selected genes. GO, Gene Ontology; KEGG, Kyoto Encyclopedia of Genes and Genomes; MCODE, Molecular Complex Detection.

Numerous studies have shown that genes with higher MM and GS could be potential candidates for further research (Fuller et al., 2007; Oldham, Horvath & Geschwind, 2006; Wang et al., 2017). Hence, based on MM > 0.6 and GS > 0.6, 174 genes were identified in the darkolivegreen module, 608 genes and 15 lncRNAs (DLEU1, EPHA5-AS1, H19, LINC00284, LINC00839, LINC00921, LINC01119, LINC01213, LINC01291, LINC01616, PVT1, SCARNA9, SMAD5-AS1, STX18-AS1, TTTY15) in the brown module, 42 genes in the darkturquoise module, and 12 genes in the orangered4 module. The Metascape website was used to perform enrichment analysis on the brown module hub genes. The results showed that the brown module is mainly related to cell cycle regulation, which is in line with the original findings published using this data set (Van de Peppel et al., 2017). Besides, it has been shown that cell cycle regulation is associated with the differentiation of bone marrow MSCs (Batsali et al., 2017; Boucher et al., 2016). In the enrichment results, the column named “Description” represents detailed pathways and biological processes, while the corresponding pathway-related genes can be found in the column named “Hits” (S1). The results showed that many pathways and biological processes are related to mesenchymal cell differentiation, stem cell differentiation, and bone development. Cytoscape was then used to identify hub genes in the brown module using the MCODE plug-in, wherein the highest ranked module was selected, which had 24 nodes, 276 edges, and an average MCODE score of 23 (Fig. 7).

### Gene expression analysis of Phase I group

**Figure 8:**
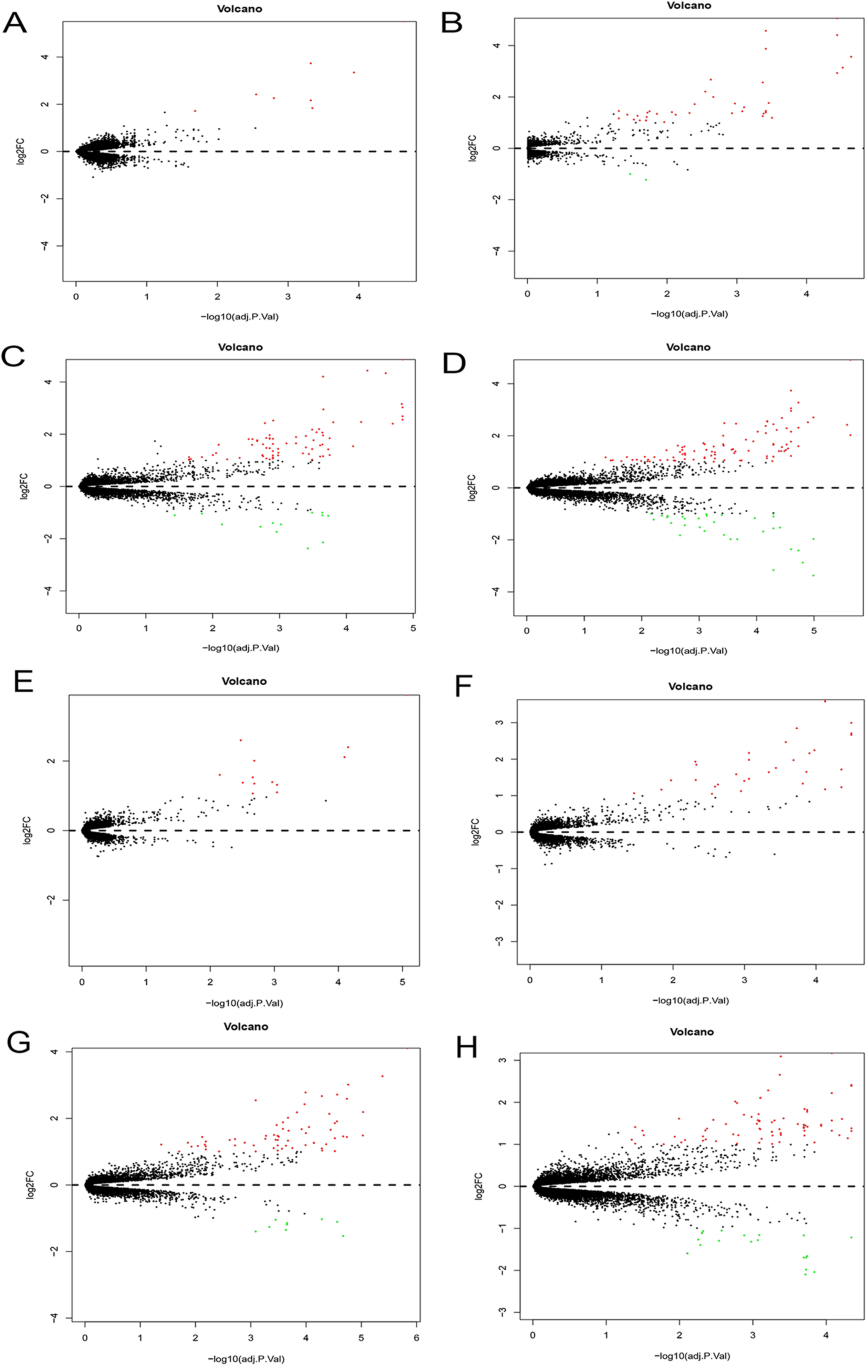
Differential expression analysis. Volcano plots were used for the visualization of the relationship between |log2FC| and statistical significance. (A–D) Volcano plots showing differential expression results from the comparison of bone marrow MSCs during AD differentiation: (A) 0.5 h vs. 0 h, (B) 1 h vs. 0 h, (C) 2 h vs. 0 h and (D) 3 h vs. 0 h. (E–H) Represent the volcano plots showing differential expression results from the comparison of bone marrow MSCs during OS differentiation: (E) 0.5 h vs. 0 h, (F) 1 h vs. 0 h, (G) 2 h vs. 0 h and (H) 3 h vs. 0 h. Red points represent upregulated genes (log2FC > 1 and *P* < 0.05), while the blue points represent downregulated genes (log2FC < −1 and *P* < 0.05). MSCs, mesenchymal stem cells; AD, adipogenic, OS: osteogenic.

The Phase I group included 30 bone marrow MSCs samples during OS and AD differentiation. The bone marrow MSCs were induced to differentiate for 0.5, 1, 2 and 3 h, respectively. Each time point was then compared with the 0 h samples to obtain DEGs. The volcano plots of OS and AD differentiation were created using the R software (Fig. 8).

### Hierarchical clustering of DEGs

**Figure 9:**
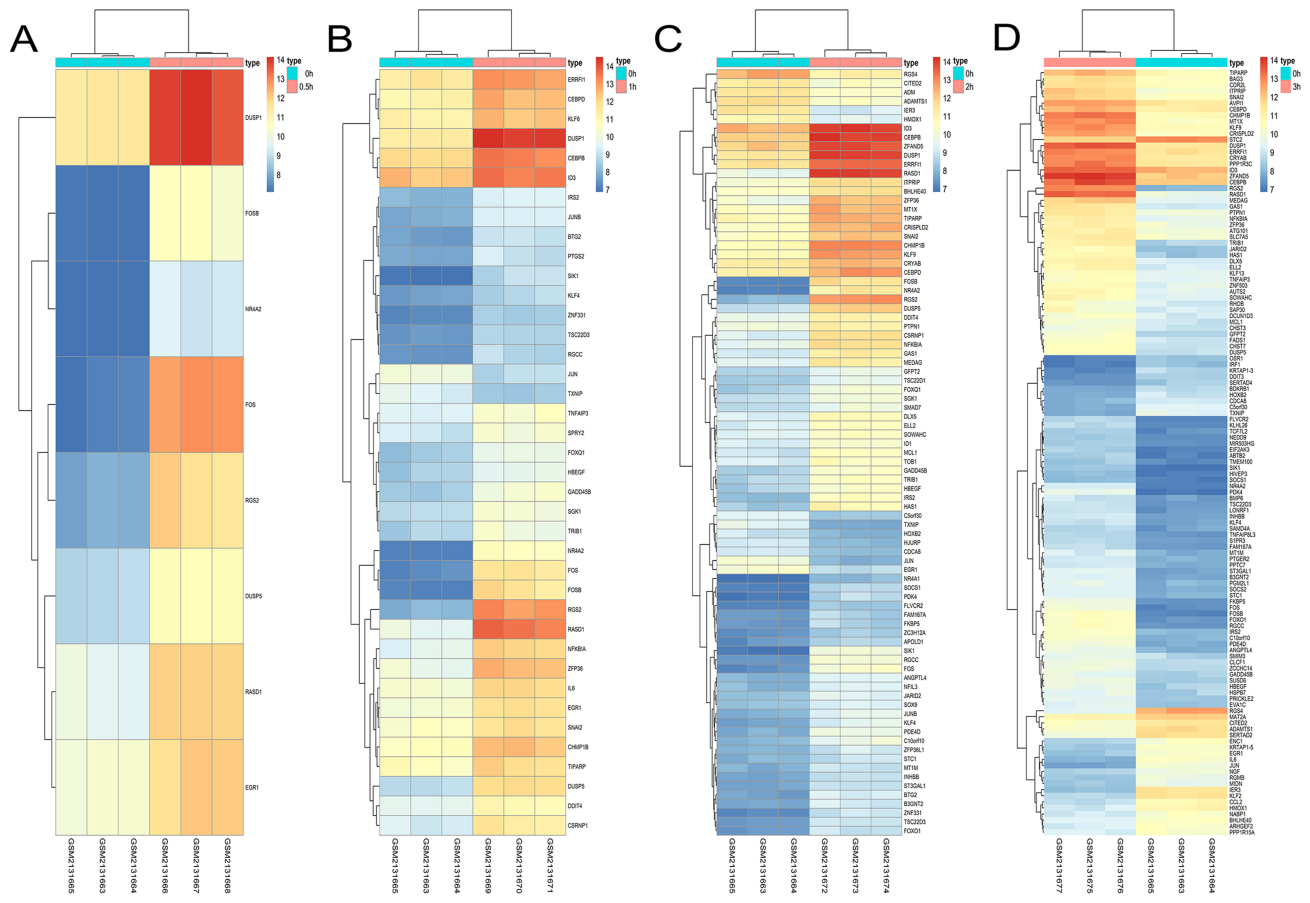
Hierarchical clustering of DGEs during AD differentiation of bone marrow MSCs. (A–D) Show the hierarchical clustering of DGEs between: (A) 0.5 h vs. 0 h, (B) 1 h vs. 0 h, (C) 2 h vs. 0 h and (D) 3 h vs. 0 h. Red and blue indicate the relative expression as indicated in the scale bars shown on the right of each figure. Each column corresponds to one sample and each row corresponds to one gene. TYPE represents the groups of samples. DGEs: differentially expressed genes, AD: adipogenic.

**Figure 10:**
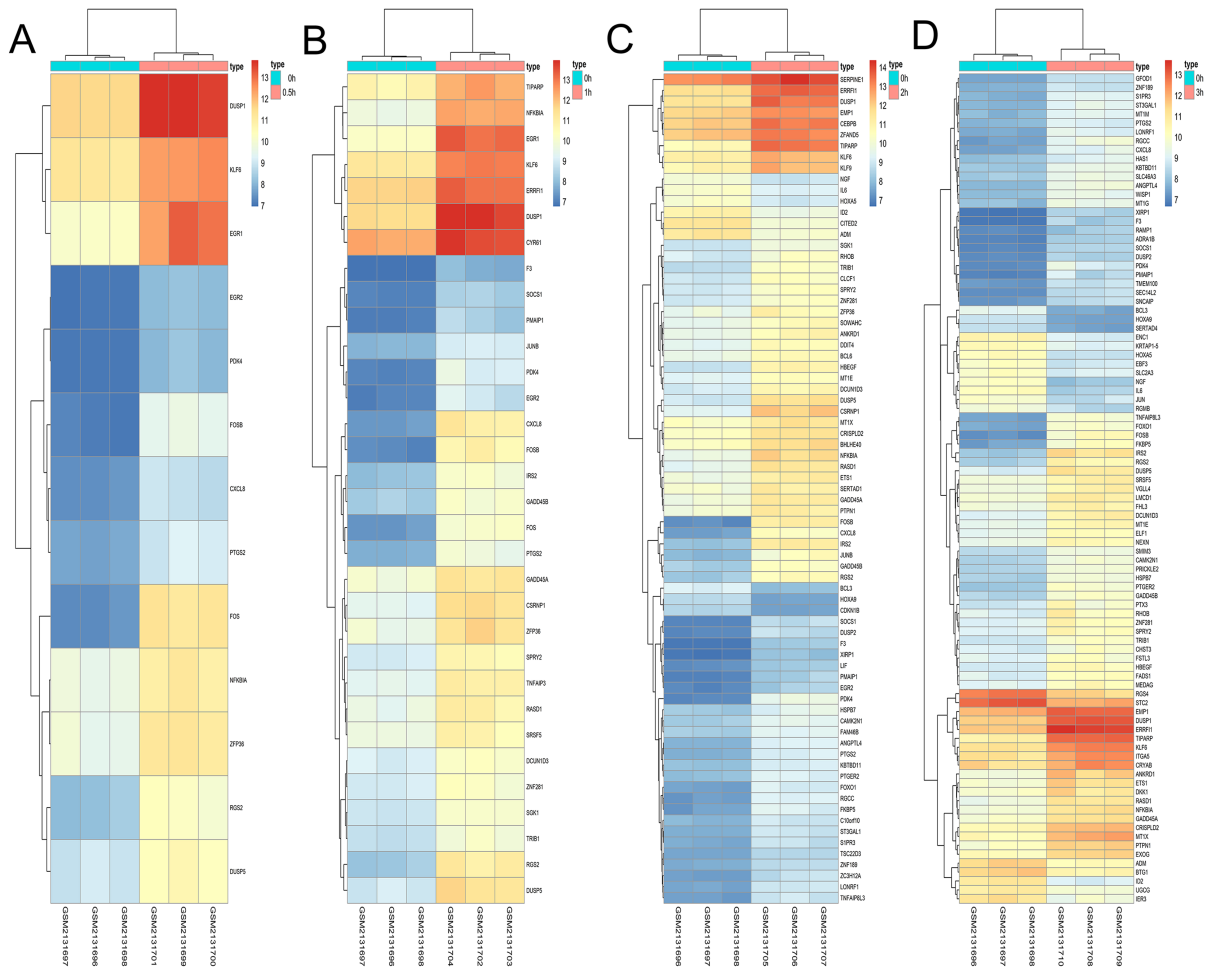
Hierarchical clustering of DGEs during OS differentiation of bone marrow MSCs. (A–D) Show the hierarchical clustering of DGEs between: (A) 0.5 h vs. 0 h, (B) 1 h vs. 0 h, (C) 2 h vs. 0 h and (D) 3 h vs. 0 h. Red and blue indicate the relative expression as indicated in the scale bars shown on the right of each figure. Each column corresponds to one sample and each row corresponds to one gene. TYPE represents the groups of samples. DGEs: differentially expressed genes, OS: osteogenic.

A total of 197 DEGs were screened, including 117 upregulated and 31 downregulated DEGs during AD differentiation, and 98 upregulated and 21 downregulated DEGs during OS differentiation (S2). Among these, in the 0.5 h vs 0 h comparison, eight genes were upregulated after 0.5 h of AD differentiation and 13 genes were upregulated after 0.5 h of OS differentiation. In the 1 h vs 0 h group, 37 upregulated and two downregulated genes were associated with AD differentiation, while 32 differentially upregulated genes were associated with OS differentiation. In the 2 h vs 0 h comparison, 72 and 13 genes were upregulated and downregulated, respectively, after 2 h of AD differentiation, while 66 upregulated and 9 downregulated genes were differentially expressed after 2 h of OS differentiation. In the 3 h vs 0 h comparison, 94 upregulated and 32 downregulated genes were associated with AD differentiation, while 74 upregulated and 19 downregulated genes were associated with OS differentiation. Hierarchical clustering of the DEGs during OS differentiation and AD differentiation was performed using the R software (Figs. 9 and 10).

### Identification of genes involved in the differentiation of bone marrow MSCs

**Figure 11:**
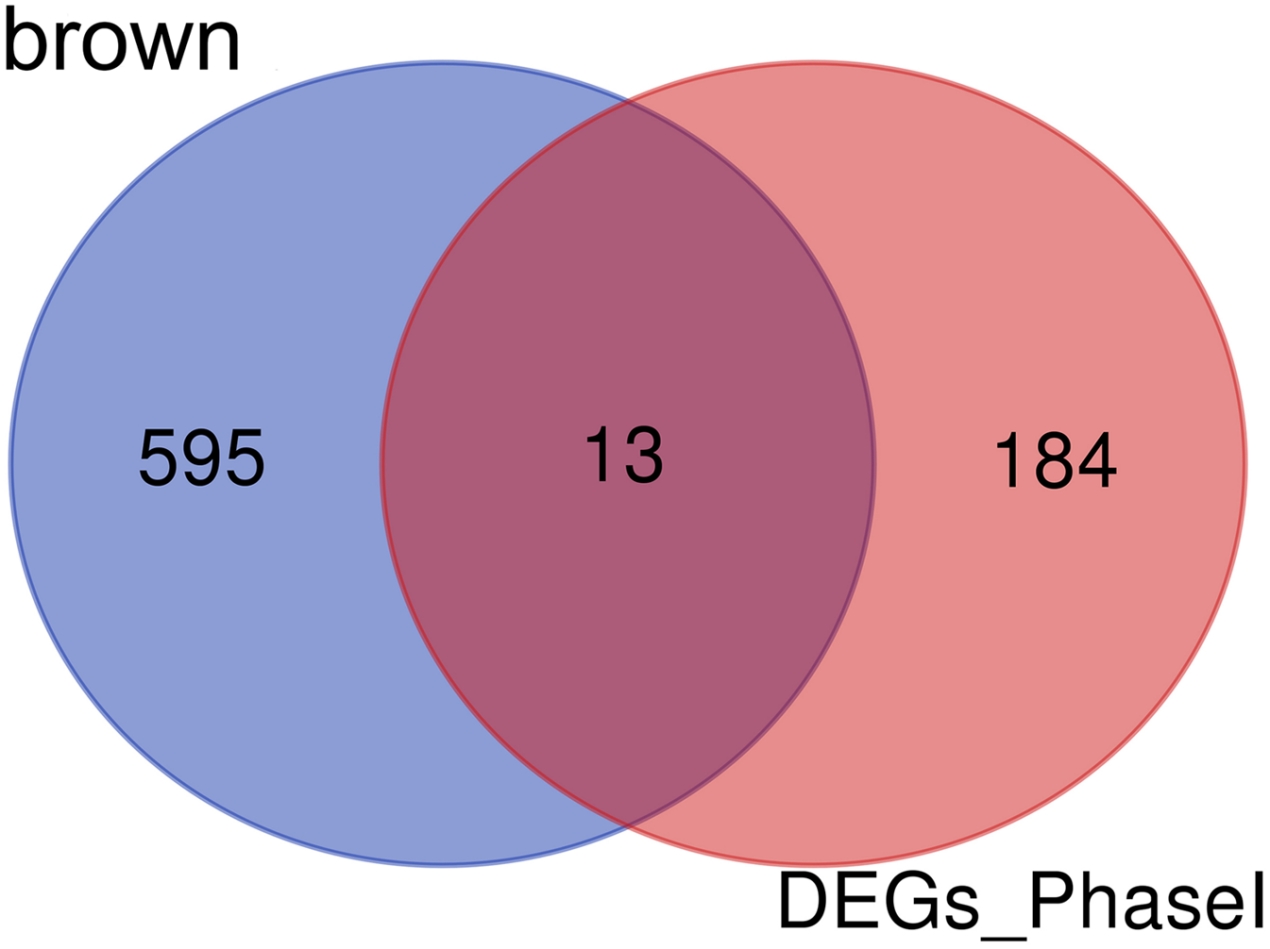
Common genes between the brown module and differential expression. The Venn diagram showing similarities between the brown module genes and DGEs during Phase I differentiation. Only 13 genes were common between DGEs during Phase I and the brown module. DGEs: differentially expressed genes.

Enrichment analysis showed that the brown module was associated with the differentiation of bone marrow MSCs. Interestingly, a set of 13 candidate hub genes were common between the brown module genes and the DEGs from Phase I (Fig. 11).

### Transcription factors and miRNAs screening

**Figure 12:**
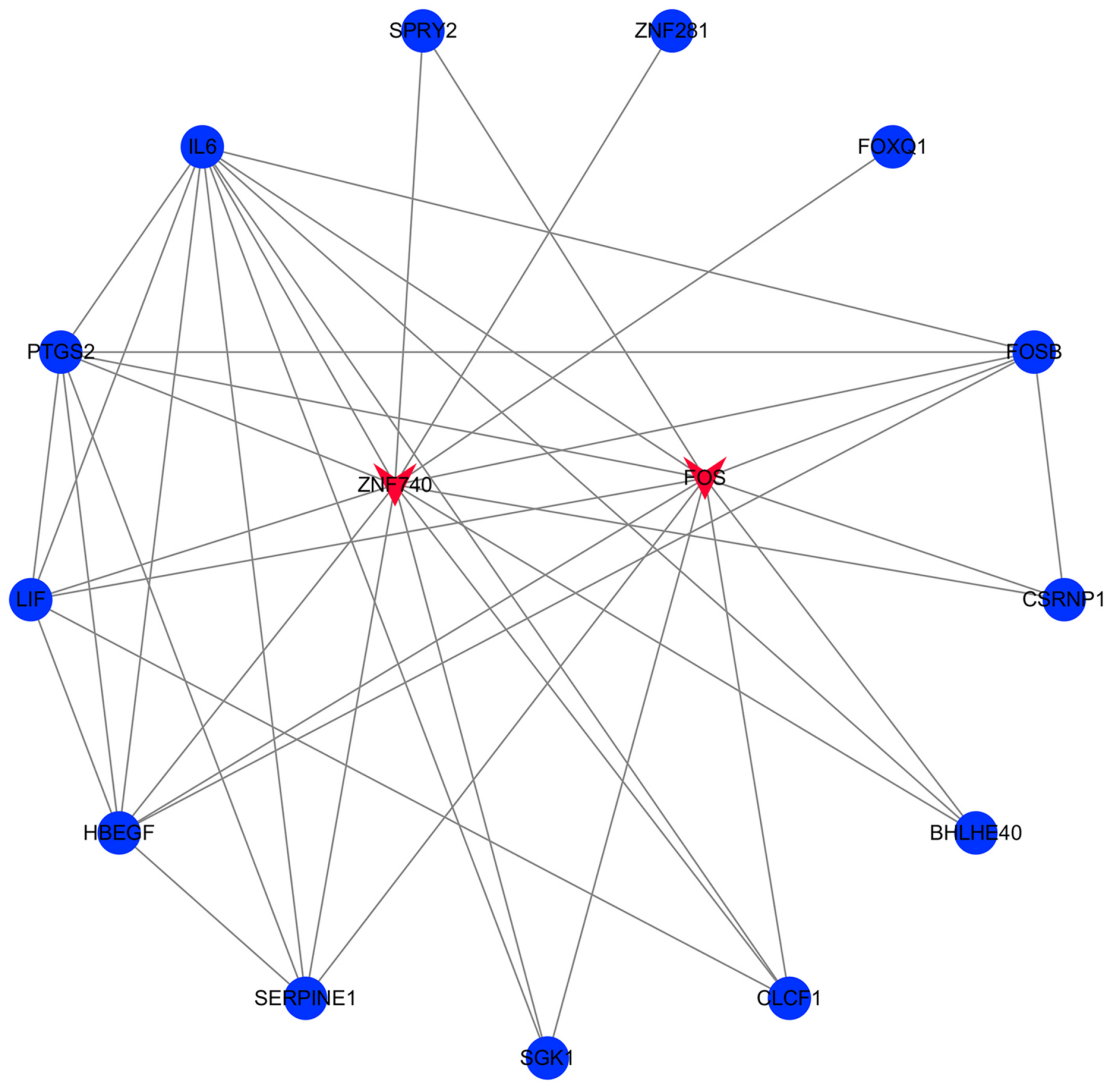
Transcription factor gene regulatory network analysis. Blue circle represents hub genes. Red V represents transcription factors.

**Figure 13:**
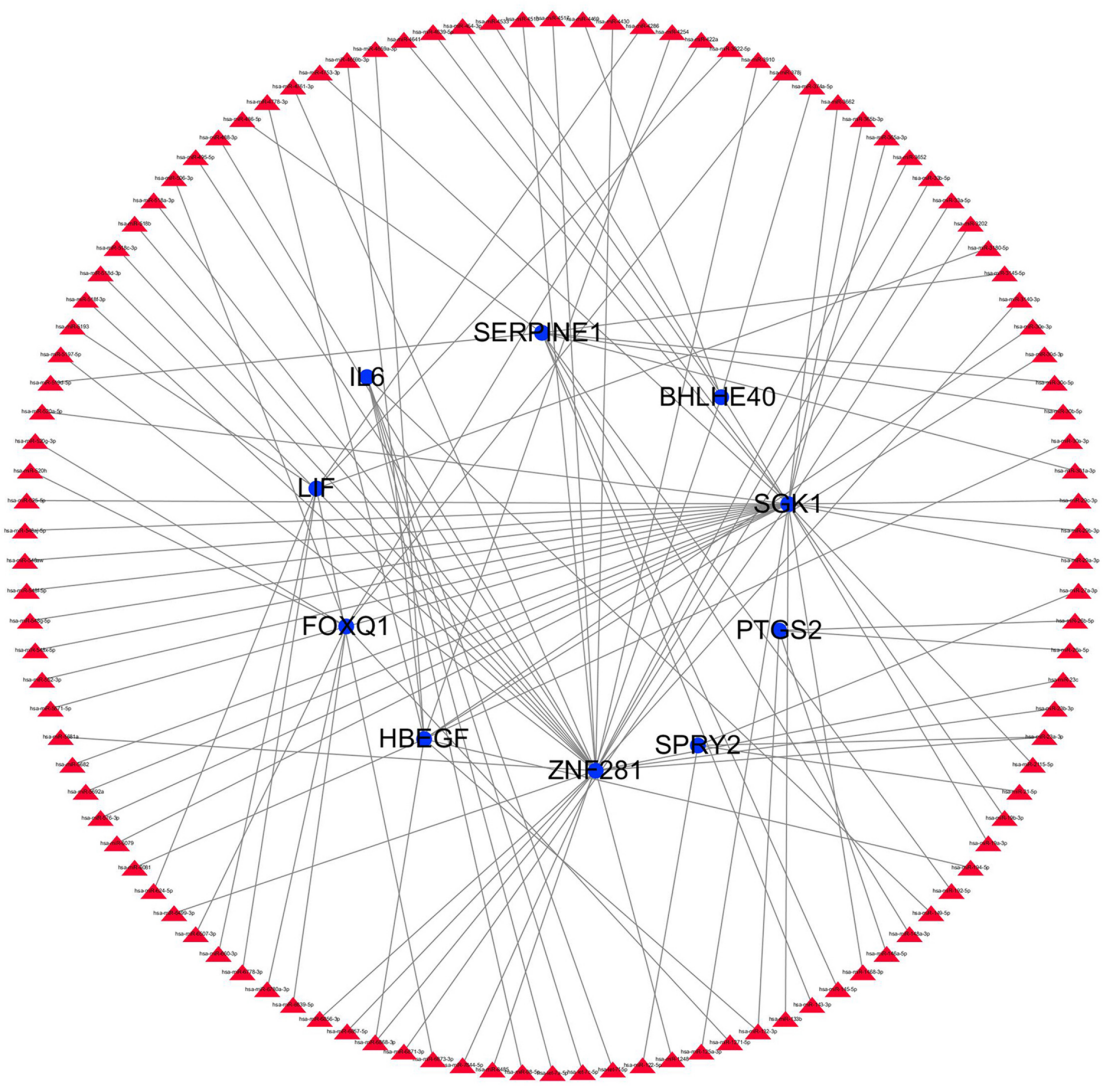
miRNA gene regulatory network analysis. Blue circle represents hub genes. The red triangle represents miRNAs.

The transcription factor regulatory network was constructed and visualized using Cytoscape software. The network included 2 transcription factors, (ZNF740, FOS), and the above-mentioned 13 candidate hub genes (Fig. 12). ZNF740 and FOS were connected with 11 and 13 hub genes, respectively. Similarly, the miRNA regulatory network was also constructed for the hub genes using TargetScan, miRTarBase and miRDB databases (Fig. 13; Table 1).

**Table 1:** The miRNA regulatory network was constructed using TargetScan, miRTarBase and miRDB databases. A total of 110 miRNAs were identified using public miRNA databases.

Gene	miRNA		
BHLHE40	hsa-miR-374a-5p	hsa-miR-8485	hsa-miR-4469
	hsa-miR-454-3p		
FOXQ1	hsa-miR-378j	hsa-miR-6507-3p	hsa-miR-6839-5p
	hsa-miR-422a	hsa-miR-6780a-3p	hsa-miR-520h
	hsa-miR-1271-5p	hsa-miR-520g-3p	hsa-miR-506-3p
HBEGF	hsa-miR-4659a-3p	hsa-miR-6868-3p	hsa-miR-4659b-3p
	hsa-miR-30a-3p	hsa-miR-30e-3p	hsa-miR-4254
	hsa-miR-194-5p	hsa-miR-4778-3p	hsa-miR-6081
	hsa-miR-30d-3p	hsa-miR-132-3p	
IL6	hsa-miR-149-5p	hsa-let-7f-5p	hsa-let-7a-5p
	hsa-let-7c-5p	hsa-miR-98-5p	
LIF	hsa-miR-3180-5p	hsa-miR-624-5p	hsa-miR-4286
	hsa-miR-660-3p	hsa-miR-6778-3p	hsa-miR-5193
	hsa-miR-3922-5p	hsa-miR-6873-3p	
PTGS2	hsa-miR-146a-5p	hsa-miR-26b-5p	hsa-miR-26a-5p
	hsa-miR-132-3p		
SERPINE1	hsa-miR-30c-5p	hsa-miR-3145-5p	hsa-miR-148a-3p
	hsa-miR-301a-3p	hsa-miR-519d-5p	hsa-miR-143-3p
	hsa-miR-30b-5p	hsa-miR-486-5p	hsa-miR-192-5p
	hsa-miR-145-5p		
SGK1	hsa-miR-576-3p	hsa-miR-2115-5p	hsa-miR-5682
	hsa-miR-5692a	hsa-miR-4641	hsa-miR-125a-3p
	hsa-miR-3662	hsa-miR-548aw	hsa-miR-548x-5p
	hsa-miR-1468-3p	hsa-miR-133b	hsa-miR-3202
	hsa-miR-19a-3p	hsa-miR-4753-3p	hsa-miR-29c-3p
	hsa-miR-19b-3p	hsa-miR-6079	hsa-miR-548aj-5p
	hsa-miR-6871-3p	hsa-miR-4639-5p	hsa-miR-29b-3p
	hsa-miR-520a-5p	hsa-miR-4533	hsa-miR-548f-5p
	hsa-miR-525-5p	hsa-miR-5571-5p	hsa-miR-29a-3p
	hsa-miR-365b-3p	hsa-miR-548g-5p	hsa-miR-552-3p
	hsa-miR-365a-3p		
SPRY2	hsa-miR-23a-3p	hsa-miR-122-5p	hsa-miR-21-5p
	hsa-miR-27a-3p		
ZNF281	hsa-miR-518a-3p	hsa-miR-6499-3p	hsa-miR-23a-3p
	hsa-miR-3140-3p	hsa-miR-6868-3p	hsa-miR-1248
	hsa-miR-4430	hsa-miR-3910	hsa-miR-6856-3p
	hsa-miR-4761-3p	hsa-miR-6857-5p	hsa-miR-5681a
	hsa-miR-518b	hsa-miR-495-5p	hsa-miR-23c
	hsa-miR-33a-5p	hsa-miR-23b-3p	hsa-miR-5197-5p
	hsa-miR-518f-3p	hsa-miR-451b	hsa-miR-7844-5p
	hsa-miR-4517	hsa-miR-488-3p	hsa-miR-518d-3p
	hsa-miR-518c-3p	hsa-miR-3652	hsa-miR-33b-5P

**Note:**

DEGs, differentially expressed genes; miRNA, microRNAs.

### Hub genes validation

Differentially expressed genes analysis was performed on the validation data set to confirm the identified hub genes (Table 2). Four of the hub genes (FOSB, CLCF1, LIF and SERPINE1) were not present in the validation data set. Four of the remaining genes, including IL6, HBEGF, FOXQ1 and SGK1, changed significantly during the early phase of differentiation (after 3 h of differentiation, |log2FC| > 1). While IL6 was significantly downregulated, HBEGF, FOXQ1 and SGK1 were significantly upregulated after 3 h of differentiation.

**Table 2:** Validate the identified hub genes. Data from the ArrayExpress database was used to validate the expression of hub genes.

Gene	logFC	AdjP
(A) 1 h after induction of differentiation vs ‘0 h’		
FOXQ1	1.7	0.05723
IL6	0.6	0.0092375
PTGS2	0.6	0.030195
SGK1	1.3	1.53E−05
SPRY2	0.7	0.0076576
FOS	3.2	0.00000072176
(B) 3 h after induction of differentiation vs ‘0 h’		
BHLHE40	−0.9	0.000714
CSRNP1	0.6	0.002071
FOXQ1	1.9	0.001246
HBEGF	1.1	9.35E−05
IL6	−1.5	4.37E−04
SGK1	1.7	1.23E−05
ZNF281	−0.9	0.017394
FOS	2.0	0.000033236

**Note:**

logFC: log2 fold change adjP: adjusted *P*-value.

### GSEA analysis on the candidate genes

**Figure 14:**
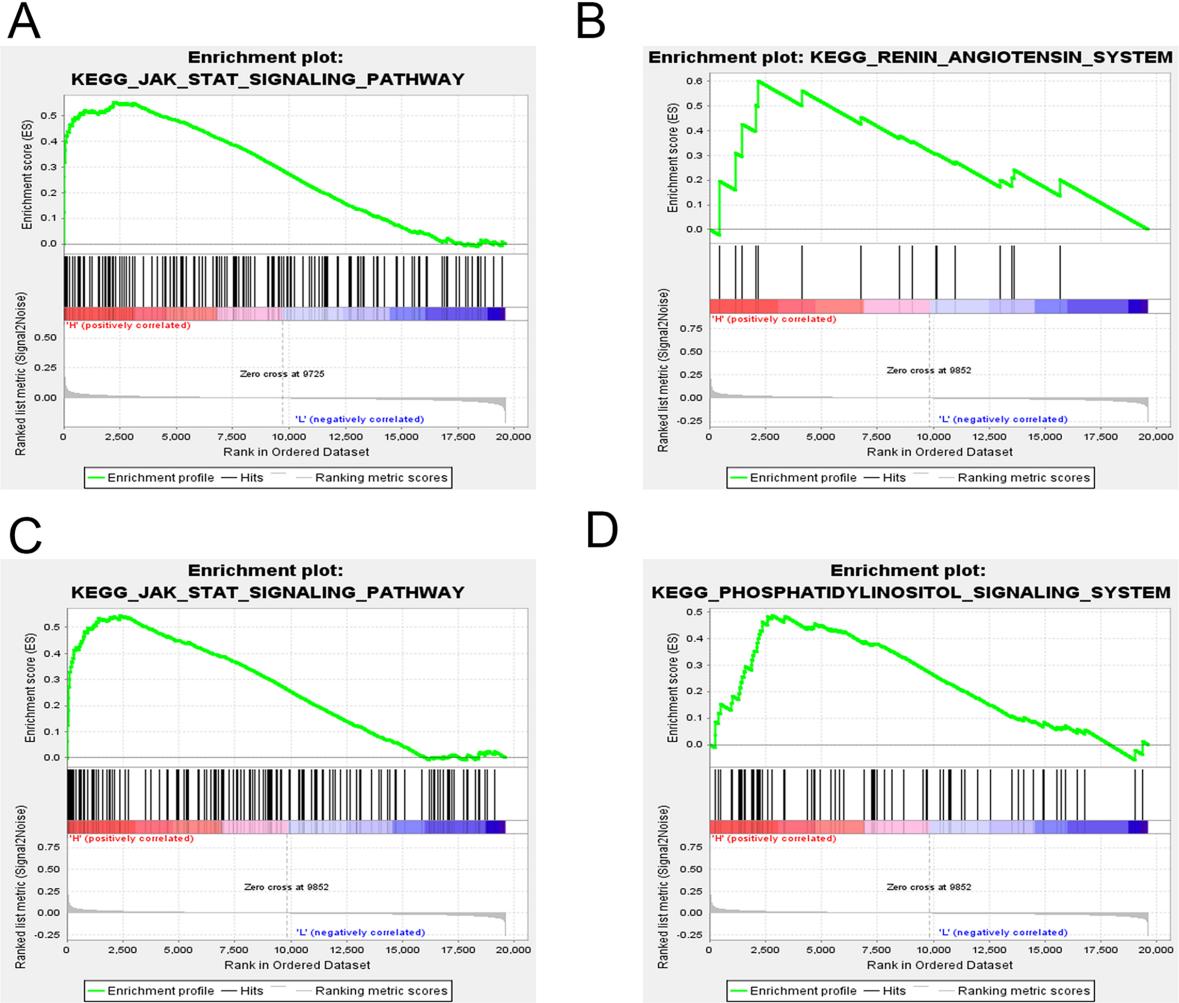
GSEA of key driver genes. (A) The gene set of JAK-STAT signaling pathway was enriched in MSCs expressing high levels of FOXQ1. Gene sets enriched in MSCs with high expression of SGK1 include: (B) RAS pathway, (C) JAK-STAT signaling pathway and (D) Phosphatidylinositol signaling system. GSEA: gene set enrichment analysis, MSCs: mesenchymal stem cells.

GSEA was applied to reveal the potential role of candidate genes (FOXQ1, SGK1) during MSC differentiation. Our results showed that the JAK-STAT signaling pathway was positively correlated with FOXQ1 expression (NES > 1.5, NOM *P*-val (nominal *P* value) < 0.05 and FDR < 0.25) (Fig. 14A). On the other hand, three gene sets were positively correlated with SGK1 expression, including the “RAS”, the “JAK-STAT signaling pathway” and the “Phosphatidylinositol signaling system” (Figs. 14B–14D).

## Discussion

Osteoporosis can increase fracture risk, which is a growing concern as the population ages. MSCs are mesoderm-derived multipotent cells that can differentiate into adipocytes, osteoblasts and chondrocytes. Transformation of MSCs into adipocytes, rather than osteoblasts, has been associated with the development of osteoporosis. Indeed, the microenvironment of the bone marrow cavity changes with age, which can lead to increase in fat cells formation and inhibition of bone formation. During this process, adipose tissue gradually replaces bone tissue, leading to osteoporosis. Therefore, understanding the molecular mechanisms of osteogenesis and MSC differentiation is particularly imperative. In this study, we used WGCNA to extract gene modules that are associated with OS and AD differentiation of bone marrow MSCs, and to identify potential biomarkers and therapeutic targets of osteoporosis.

First, we constructed a co-expression network containing 22 modules, which were then compared with DEGs during Phase I (0–3 h), Phase II (6–24 h), Phase III (48–96 h), AD and OS differentiation of MSCs. The brown module was highly enriched in genes involved in cell cycle-related pathways and other processes related to MSC differentiation, such as mitotic nuclear division (Dudakovic et al., 2014), DNA repair (Dudakovic et al., 2014), and chromosome maintenance (Oktar et al., 2011). Interestingly, the enrichment results showed that numerous genes were related to osteoblast differentiation, bone mineralization, bone remodeling, bone resorption, and skeletal system development. Then, we analyzed 608 genes that were selected based on the parameters of MM and GS to obtain hub genes with the highest scores (MM > 0.85, GS > 0.85). Among these genes, the NRG1 gene has been shown to mediate cell–cell signaling and play a critical role in the growth and development of multiple organ systems. Besides, NRG1 was shown to be upregulated by Wnt3a during Wnt3a-induced osteoblast differentiation in primary human MSCs (Jullien et al., 2012). On the other hand, ITGA2 was shown to induce the expression of WNT5A to promote OS differentiation of human MSCs (Olivares-Navarrete et al., 2011). SERPINB2 is a TGF-β-responsive gene that plays a negative regulatory role in the differentiation of human bone stromal cells (Elsafadi et al., 2017). PRKCA, another hub gene, was demonstrated to regulate bone architecture and osteoblast activity (Galea et al., 2014). FAM20C encodes a family member of secreted protein kinases, which can bind calcium and phosphorylate proteins involved in bone mineralization (Liu et al., 2018). RBPJ, a primary nuclear mediator of Notch signaling, has also been associated with osteogenesis (S3) (Wang et al., 2010).

In order to screen for candidate genes that can serve as potential biomarkers or therapeutic targets, the Phase I samples, associated with the brown module, were compared with the control samples, which led to the identification of 119 DEGs related to OS differentiation and 148 DEGs that were associated with AD differentiation. The identified DEGs were then compared with the brown module, which led to the identification of 13 common genes, including BHLHE40, FOSB (Schneider et al., 2017), CSRNP1, HBEGF (Li et al., 2019a), CLCF1 (Nahle et al., 2019), SPRY2 (Schneider et al., 2017), IL6 (Munir et al., 2017), PTGS2 (Feigenson et al., 2017), SGK1, FOXQ1, LIF (Huat et al., 2014), SERPINE1 (Karbiener et al., 2011) and ZNF281. Of interest, the previously identified OS differentiation-related genes FOSB, SPRY2 and PTGS2 were upregulated, while HBEGF, CLCF1 and LIF were downregulated. In addition, IL6 and SERPINE1, involved in AD differentiation of bone marrow MSCs, were downregulated. Among the identified candidate genes, BHLHE40, CSRNP1, SGK1, FOXQ1 and ZNF281 could be novel potential targets related to OS and AD MSC differentiation. Moreover, 110 miRNAs and 2 transcription factors (ZNF740, FOS) were identified using public databases as candidate upstream regulators of the network’s hub genes. We found that both ZNF740 and FOS genes were differentially expressed during Phase I of differentiation, while only FOS was upregulated during OS and AD differentiation. Besides, FOS was also a member of the brown module and was differentially expressed in the validation data set. The transcription factor FOS belongs to the FOS gene family, which has been described as a regulator of cell proliferation, differentiation and transformation. On the other hand, another data set of MSC differentiation was used to validate four candidate genes with significant differential expression (IL6, HBEGF, FOXQ1 and SGK1). Finally, GSEA was performed to identify candidate pathways associated with FOXQ1 and SGK1 expression, which showed that FOXQ1 is associated with JAK-STAT signaling pathway, while SGK1 is mainly associated with the RAS. Studies have shown that inhibition of the JAK-STAT signaling pathway promoted OS differentiation (Levy et al., 2010). The RAS regulated ossification and angiogenesis of bones, as well as OS differentiation (Durik, Sevá Pessôa & Roks, 2012). On the other hand, the phosphatidylinositol signaling system could regulate the OS and AD differentiation of bone marrow MSCs (Song et al., 2017).

SGK1 is a member of the PI3-kinase pathway, which regulates cell survival, proliferation and differentiation (Lang et al., 2006). In addition, we observed that genes in the darkolivegreen module were associated with cell cycle arrest, cell proliferation and apoptotic processes, but also were related to PI3-kinase and MAPK signaling pathway that could also participate in osteoblast differentiation. In fact, the PI3-kinase signaling pathway is downstream to the JAK-STAT and RAS signaling pathways. Therefore, we may infer that the brown and the darkolivegreen modules could be complementary, or common, with different co-expression patterns. Among the genes associated with the PI3K pathway in the darkolivegreen module, ESR1 encodes the estrogen receptor ERα that regulates the classic estrogen signaling pathway. Studies have shown that ERα deficiency inhibited OS differentiation and promoted adipocytic differentiation of bone MSCs (Li et al., 2019b). In breast cancer, ERα expression was associated with mTORC1-mediated phosphorylation of SGK1-Ser422 (Hall et al., 2012). These results indicate a possible interaction between ESR1 and SGK1 during MSC differentiation. In addition, SRC, a non-receptor tyrosine kinase, is a key signaling molecule in bone metabolism that enhances OS differentiation through phosphorylation of Osterix (Choi et al., 2015). Studies have shown that in some human diseases, SGK1 was the key mediator of SRC-induced transformation (Ma et al., 2019). So, we may assume that SRC can regulate the expression of SGK1 during MSC differentiation. In conclusion, our study suggests that FOXQ1 and SGK1 may regulate the OS and AD differentiation of bone marrow MSCs through these signaling pathways, which requires further experimental validation in the future.

## Conclusion

In summary, WGCNA and DEGs analysis were used in this study, which showed that correlated genes in the brown module could be critical for the differentiation of bone marrow MSCs. A total of two transcription factors and two hub genes, which are potential targets of 110 miRNAs, were associated with OS and AD differentiation of MSCs. These key drivers may serve as potential biomarkers for the diagnosis and prognosis of osteoporosis, which need to be validated by more studies in the future.

## Supplemental Information

10.7717/peerj.8907/supp-1 Supplemental Information 1 Enrichment results of 608 genes in the brown module of the Metascape website. The column named ’Description’ includes pathways and biological processes. The column named ’Hits’ includes genes involved in each term.

10.7717/peerj.8907/supp-2 Supplemental Information 2 A total of 197 DGEs were selected using the *limma* package in R software. The gene symbol of 157 upregulated DEGs and 40 downregulated DEGs are ordered by their log2FC and p-values. DGEs: differentially expressed genes.

10.7717/peerj.8907/supp-3 Supplemental Information 3 Hub genes selected by gene MM and GS. Only 24 genes were obtained with MM > 0.85 and GS > 0.85. MM: module membership, GS: gene significance.

10.7717/peerj.8907/supp-4 Supplemental Information 4 Raw data for non-normalized expression profiles.

10.7717/peerj.8907/supp-5 Supplemental Information 5 Raw data for normalized expression profiles.

10.7717/peerj.8907/supp-6 Supplemental Information 6 Gene names were used to annotate the processed data.

10.7717/peerj.8907/supp-7 Supplemental Information 7 Code for DEGs analysis. The script was written in R using the *limma* package. DGEs:differential gene expressions.

10.7717/peerj.8907/supp-8 Supplemental Information 8 Code for WGCNA. The script was written in R using the WGCNA algorithm. WGCNA: Weighted Gene Co-Expression Network Analysis.

10.7717/peerj.8907/supp-9 Supplemental Information 9 Code for hierarchical clustering using *pheatmap* package. The script was written in R using the pheatmap algorithm.

10.7717/peerj.8907/supp-10 Supplemental Information 10 Miame Checklist. 3rd party conducted the microarray experiment, this document does not need to be published EP

## Additional Information and Declarations

### Data Availability

The following information was supplied regarding data availability: Data is available at NCBI GEO: GSE80614.
