# Supplementary material for: Weighted gene correlation network analysis reveals novel biomarkers associated with mesenchymal stromal cell differentiation in early phase
Source: PeerJ. 2020 Apr 3;8:e8907. doi: 10.7717/peerj.8907 (PMC7134052; doi:10.7717/peerj.8907)
Supplement: Supplemental Information 10 — 3rd party conducted the microarray experiment, this document does not need to be published EP [file peerj-08-8907-s010.docx]

**Miame Checklist**

Part 1 Experiment description

- **hMSC differentiation**
- **Number of samples**

**WGCNA** (n = 66)

**DEGs** ( [adipogenic](javascript:;) [differentiation](javascript:;) (n = 15) , [osteogenic differentiation](javascript:;) (n = 15) )

#### experimental variables

**WGCNA** (0-3h, Phase I; 6-24h, Phase II; 48-96h, Phase III )

#### DEGs ( [adipogenic](javascript:;) [differentiation](javascript:;) (0.5h vs 0h; 1h vs 0h; 2h vs 0h; 3h vs 0h) , [osteogenic differentiation](javascript:;) (0.5h vs 0h; 1h vs 0h; 2h vs 0h; 3h vs 0h) )

Part 2 Experimental Materials

- **Raw data (supplementary material 4).** This data was provided by Professor van de Peppel J (Email : h.vandepeppel@erasmusmc.nl).
- **Normalized data** **(supplementary material 5)**

33 samples from serial number GSM2131663-GSM2131695 represent adipogenic differentiation; 33 samples from serial number GSM2131696-GSM2131728 represent osteogenic differentiation; In these samples, there are 3 biological replicates at each time point.

Part 3 Analysis

- **DEGs (supplementary material 2)**

The first column represents the gene symbol and the other columns represent the expression value of every gene. Other detailed information such as gene ID, GenBank Accession are included in the platform annotation file **(supplementary material 6)**.

- **WGCNA (supplementary material 1)**

A column described as 'Category' represents the type of enriched gene set;

A column described as 'Hits' represents several genes in brown module enriched in gene sets; Columns described as 'Description' and 'GO' represented biological processes and detailed pathway of enriched genes, respectively.

- **Other bioinformatics tools**

Version numbers, parameters, and detailed operations of Cytoscape and GSEA software are included in the manuscript.

- **Code for Differential expressed gene (DEG) analysis (supplementary material 7)**
- **Code for Weighted gene co-expression analysis (WGCNA)**

**(supplementary material 8)**

- **Code for heatmap using pheatmap package**

**(supplementary material 9)**
